# The Dynamics of Mood in Bipolar Disorder: How Mathematical Models Help Phenotype Individuals, Forecast Mood, and Clarify Underlying Mechanisms

**DOI:** 10.1007/s11920-025-01647-z

**Published:** 2025-10-27

**Authors:** Amy L. Cochran, Jimmy Vineyard

**Affiliations:** 1 Department of Population Health Sciences, University of Wisconsin-Madison, Madison, WI US; 2 Department of Mathematics, University of Wisconsin-Madison, Madison, WI US; 3 University of Wisconsin, Room 685 WARF; 610 Walnut St, Madison, WI 53726-2397, 0000-0001, 6024-796X USA

**Keywords:** Bipolar disorder, Dynamics, Mood, Phenotype, Models, Forecast

## Abstract

**Purpose of Review:**

Mood in bipolar disorder (BP) fluctuates in complex and unpredictable ways that resist simple explanation. To capture this complexity, researchers have turned to modeling mood dynamics. This review organizes the recent literature around three key questions: How can modeling help phenotype BP? Can models accurately predict future mood? And can modeling clarify mechanisms underlying mood instability?

**Recent Findings:**

Models of mood dynamics carry clinically relevant information beyond standard measures, differentiating BP from other disorders and reflecting treatment response and impairment. Yet their ability to forecast mood tends to deteriorate within days, raising the possibility that fleeting predictability may be intrinsic to BP, not simply a technical limitation. Mood also appears to be best represented by two or more continuous latent dimensions, with states like mania and depression appearing as loosely defined regions. However, how mood unfolds over time remains poorly understood, particularly the relative influence of internal dynamics versus external triggers.

**Summary:**

Looking ahead, two priorities stand out: applying these models in real time to support clinical and research decisions, and validating them systematically through standardized forecasting tasks and shared longitudinal datasets integrating mood and physiological data. Together, these steps can help turn modeling into a practical tool for understanding and managing mood in BP.

## Introduction

In bipolar disorder (BP), mood fluctuations are far more erratic and unpredictable than clinical terms like “episodes” and “cycling” suggest. These terms, while clinically useful, imply clear boundaries between moods and evoke regular alternation between states. But longitudinal data reveal a reality that is more complex: manic and depressive symptoms often overlap, vary continuously, change unpredictably, and defy easy classification. Over the past three decades, researchers have increasingly turned to mathematical models to better capture this complexity. These models are applied to repeated measures of mood, behavior, or physiology and span a wide range of approaches.

As the diversity and complexity of these models have grown, their vocabulary, assumptions, or clinical relevance have become challenging to navigate. This review clarifies this landscape by organizing the recent literature around three central questions: How can modeling help phenotype individuals with BP? Can models accurately predict future moods? And can modeling uncover the mechanisms driving mood instability? We highlight key advances, remaining challenges, and the steps needed to ensure these models become useful for both clinical care and scientific discovery.

## To Phenotype

A common use of mathematical models in BP research is to describe individuals based on how their mood fluctuates over time. This is a form of clinical *phenotyping* and is accomplished by fitting models to an individual’s mood data and extracting parameters that summarize their mood dynamics. These parameters help distinguish diagnostic groups, predict outcomes like recurrence or suicidality, or quantify treatment effects such as response to lithium.

This strategy is particularly valuable for teasing apart conditions that share similar symptoms, like BP and borderline personality disorder. Based on average levels of daily mood ratings and their variances, BP falls somewhere between borderline personality disorder and nonclinical controls, incorrectly suggesting that BP is a milder version of borderline personality disorder rather than a distinct condition [1, 2]. To address this, Pulcu et al. fit a model to describe an individual’s mood dynamics, focusing on two distinct parameters [2]. The first parameter, *volatility*, captures slow, persistent shifts in mood over several days, akin to the gradual change in temperature from summer to winter. The second, *noise*, captures erratic, short-term fluctuations, akin to the rise and fall between daytime heat and nighttime cold. This model-based decomposition revealed that BP is characterized by high volatility, borderline personality disorder by high noise, and controls by low levels of both. Their work showed that the two mood disorders can be meaningfully distinguished by *how* mood changes, not just by *how much*. Notably, the model also revealed that individuals with BP showed increased volatility in positive mood when on lithium, challenging the assumption that lithium simply stabilizes mood, suggesting instead that it helps individuals shift into positive emotional states.

This example illustrates a growing view of BP as a disorder defined not only by its extreme moods but by the way mood shifts over time. This perspective has motivated efforts to identify individuals with more unstable trajectories [3], an area where modeling approaches have proven especially useful. Prisciandaro et al. analyzed clinician-rated symptom data from over 3,900 individuals collected quarterly in the STEP-BD study, identifying three empirically-derived mood states: euthymic, depressed, and mixed [4]. The mixed state, characterized by co-occurring manic and depressive symptoms, was less common and less stable than the euthymic or depressed state, and was associated with a prior history of rapid cycling and substance use. These findings confirmed earlier observations in Cochran et al., which relied on retrospective data from a smaller sample (*n* = 209) to identify stable, depressive, and rapid-cycling trajectories in individuals with BP I, with the rapid-cycling group exhibiting greater functional impairment than the other groups [5].

Complementary evidence reinforces the idea that mood variability itself can be a marker of illness severity. Sperry et al. measured within-person variability on self-report scales of depression, mania, and anxiety, collected bimonthly over more than a decade in the Prechter Longitudinal Study of BP [6]. Individuals with BP were more likely than psychiatric or nonclinical controls to show moderate or high variability across all three symptom domains. Importantly, variability was negatively associated with how well someone was functioning emotionally, even after accounting for their diagnosis. These findings build on Sperry’s earlier work: variability along with more abrupt shifts in daily ratings of mood could predict onset of new bipolar-spectrum disorders three years later [7], and were linked to higher bipolar-spectrum traits, even after accounting for overall mood levels [8]. Additionally, people higher on these traits were linked to sharper rises in negative ratings during moments of stress [9].

While many modeling approaches in BP focused on mood, an alternative perspective centers on functional change over time. De la Fuente-Tomás et al. classified individuals with BP into five stages using markers such as clinical severity, functional impairment, cognitive performance, physical comorbidities, and quality of life [10]. Patients were tracked across three years, and their movement between stages was analyzed descriptively, showing that most individuals remained stable or moved only one stage over time, with progression linked to worse outcomes. While this work does not involve modeling in a formal sense, it naturally invites it: these discrete transitions could be represented using models, where transition probabilities between stages are estimated over time.

As one final illustration of phenotyping, Holmes et al. provided an early demonstration that mathematical modeling can provide insight into who has responded to treatment [11]. In a small study of 14 individuals undergoing a novel cognitive therapy for BP, they used daily depressive ratings to model mood dynamics before and after treatment. Most patients showed reduced time spent in depressive states and simpler longitudinal trajectories after treatment, showing how models can be used to measure treatment success.

### To Forecast

Another key goal of modeling in BP is to forecast mood. The basic question is: *Can past data tell us what a person’s mood will be next?* One early attempt to forecast mood in BP was made by Moore et al., who wanted to predict next-week depression scores based on weekly self-ratings [12]. They compared several approaches, including a persistence model (assuming next week’s mood would match this week’s) and a sample mean model (predicting the person’s average mood each week). These simple approaches did surprisingly well: the persistence model outperformed more sophisticated approaches, and even the sample mean outperformed the persistence model in about half of the cases. In follow-up work, Moore et al. reinforced their conclusions, as they more carefully searched for evidence to support moving away from these simple models. Again, they found more sophisticated models had no advantage over the simpler linear methods in forecasting of weekly depressive data [13]. In other words, simple models, such as assuming no change or reverting to one’s average mood, often rivaled or outperformed complex approaches, hinting at inherent limits to short-term mood prediction based on past mood alone.

Since mood can change meaningfully within just a few days, it is not surprising that weekly ratings are difficult to forecast. Daily ratings, by contrast, might seem more promising. Yet, when Ortiz et al. analyzed self-rated moods collected twice a day over 3 months from 30 individuals with BP and 30 nonclinical controls, they found that the best predictions came from simple models that use only the most recent rating [14]. Similarly, Sagorac Gruichich et al. reached the same conclusion using daily ratings of mania and depression from 43 individuals with BP [15]. In a larger study, Busk et al.. analyzed daily mood ratings from 84 individuals with BP [16]. Their best-performing model used *four* prior days of mood data to predict the next day’s score, achieving an R² value of 0.51 and a root mean square error of 0.32 (on a 7-point scale). However, as the prediction window extended to seven days, accuracy declined, and forecasts regressed toward the person’s average mood. Together, these studies reinforce earlier conclusions: even with daily ratings, mood fluctuations remain challenging to forecast beyond a few days, and simple approaches often yield the most reliable results.

Given how challenging it is to forecast mood even a few days ahead, some researchers have turned to an alternative strategy rooted in dynamical systems theory [17, 18]. This perspective often treats mood as part of a system that can transition between qualitatively distinct states, such as mania, depression, or euthymia. From this view, the goal is not to forecast the exact mood, but to detect when the system is becoming unstable and approaching a transition point. In theory, such transitions are often preceded by early warning signals—like rising variability and greater temporal correlation—which reflect a loss of system stability. Bos et al. applied this approach using mood ratings collected five times daily from individuals with BP over the course of four months [19]. They tested whether these indicators of instability could anticipate shifts into depression or mania. While such signals occasionally preceded transitions, they were also observed in individuals who remained stable, and often failed to appear when a transition occurred. The authors concluded that, although conceptually promising, the clinical utility of early warning signals “is yet far away.” This concern echoes a broader critique by Helmich et al.., who argue that applying such indicators in clinical settings will require deeper theoretical justification and more reliable measurement techniques [20].

Applying another tool from dynamical systems theory, Ortiz et al. quantified the predictability of mood in BP using the Lyapunov exponent [21]. The core idea is to compare two similar starting points in time and observe how similarly mood unfolds afterward. A larger exponent means small initial differences rapidly grow, reflecting greater unpredictability. Although individuals with BP and their first-degree relatives had lower exponents than nonclinical controls, suggesting greater predictability, the overall predictability remained low. The authors concluded that the window for accurately forecasting mood in BP is “inevitably short.”

With mood showing limited forecasting power on its own, attention has turned to whether changes in sleep, activity, and circadian rhythms might improve forecasting. Analyzing 90 days of daily self-reports from individuals with BP, Ortiz et al. found that self-rated energy levels outperformed self-rated mood or anxiety ratings in predicting upcoming depressive episodes [22]. Building on this, they conducted a year-long study of 127 individuals with BP wearing smart rings to continuously monitor activity and sleep [23]. Using spectral analysis, they examined how the frequency distribution of sleep and activity measurements changed over time, flagging sudden changes in the distribution for each measurement as possible early indicators of depressive episodes. Their best-performing measurements of activity and sleep correctly identified depressive episodes with 80% sensitivity and 87% specificity. Importantly, activity-based measurements signaled upcoming depressive episodes up to seven days in advance, compared to four days for sleep-based indicators. In a parallel study, the research team also used their spectral analysis approach to predict upcoming hypomanic episodes, achieving 94% sensitivity and 90% specificity [24]. In this case, frequency change in sleep was the earliest indicator of an upcoming hypomanic episode, signaling up to three days in advance.

A complementary approach was taken by Jakobsen et al.., who recorded wrist-based motor activity in individuals with BP and also looked for sudden shifts in activity patterns that might forecast an impending episode [25]. Using a different algorithm than Ortiz et al. [23], they compared short segments of activity data and flagged time periods where those patterns stopped looking similar to nearby segments, potential signs that a depressive or hypomanic episode was beginning. Roughly 11 days before depression and 16 days before hypomania, the algorithm flagged shifts in behavior that deviated from the person’s usual activity patterns. Combined, the findings from Jakobsen et al. and Ortiz et al. support a growing view that changes in activity can reveal early warning signs of mood episodes before mood symptoms themselves become apparent.

These next studies adopt a different perspective on forecasting. Rather than detecting changes in sleep and activity as indicators of an impending episode, they explore whether these changes might actively contribute to episode onset, and thus be used to predict it. Specifically, they focus on circadian rhythms as potential contributors to mood episodes. In a large observational cohort study, the Mood Disorder Cohort Research Consortium, Song et al. analyzed wearable data from individuals with major depressive disorder, BP I, and BP II disorder [26]. They computed transfer entropy, which measures how much knowing one signal (e.g., circadian phase) adds to the prediction of another (e.g., mood), beyond what the latter signal can predict about itself. Their findings revealed that, in BP I patients, changes in circadian phase often preceded changes in mood. In contrast, this predictive relationship was not observed in individuals with BP II, nor was the midpoint of sleep as predictive as circadian phase. This suggests that in BP I, shifts in circadian phase come first, preceding changes in mood.

Further emphasizing the role of circadian rhythms in mood forecasting, Lee et al. also analyzed wearable data from individuals with mood disorders in the Mood Disorder Cohort Research Consortium. Their models used features of sleep, activity, and circadian rhythms derived from wearable data in the prior 18 days to predict the occurrence of mood episode within the subsequent three days [27]. Prediction features included estimates of circadian phase and amplitude. These models performed exceptionally well, achieving area under the curves (AUCs) of 0.96 for mania, 0.96 for hypomania, and 0.94 for depression. Using data from the same cohort, Lim et al. focused on predicting next-day mood episodes from wearable data [28]. Notably, they employed a biologically-informed mathematical model of the circadian pacemaker that simulates core body temperature based on light exposure, from which they estimated Dim Light Melatonin Onset, a gold-standard measure of circadian phase [29]. Prediction performance was strong (AUCs of 0.98 for mania, 0.95 for hypomania, and 0.80 for depression). Remarkably, circadian phase emerged as the most influential predictor among 36 tested features, with advanced phases linked to mania, and delayed phases with depression.

Beyond circadian rhythms, a few small studies have explored whether other biological signals might forecast mood shifts. In particular, Valenza et al.. found that nonlinear features of heart rate variability, derived from electrocardiogram recordings, could predict whether or not an individual with BP would be euthymic on the next day [30]. Gentili et al.. extended this work, showing that heart rate variability before or after the current mood state carry information that improves detection of that state [31].

While our discussion has centered on studies aimed at forecasting future moods, a broader literature has aimed to predict a range of BP-related outcomes, including diagnosis of BP, current mood, future mood instability, and individual symptoms (c.f [32–45]).,. Attention is often given to data collected on voice, smartphone typing patterns, sleep, heart rate, electroencephalography (EEG), lab tests, and functional Magnetic Resonance Imaging (fMRI). While these prediction models are not always about forecasting, they are valuable because they demonstrate that clinically relevant information about BP can be extracted from data collected passively and objectively, potentially offering scalable, low-burden alternatives to self-report and clinician-administered assessments.

## To Understand

A final goal is to use mathematical models to *understand* the processes driving mood towards extreme values in BP. Every model begins with a basic choice: what, exactly, are we trying to model? These targets are often called *state* variables, quantities that evolve over time and define the system’s behavior. While this may sound like a technical detail, it is at the heart of a major divide in how researchers use models to understand BP. Models differ sharply in what the key state variables are, what they represent psychologically or biologically, and how they connect to real-world measurements. This matters, because if state variables are driving mood fluctuations, then identifying them presents us with possible targets for intervention.

Early models often relied a single state variable, where high values would signify mania, and low values would signify depression [46–49]. This simplified view has since been reconsidered, as it overlooked mixed features, which are both prevalent and clinically important. Indeed, a recent study has revisited some models of BP to explicitly incorporate mixed features [50–52], and when derived from data, state variables frequently capture mixed features [3, 4, 53, 54].

Nowadays, mood is usually described with two state variables: mania and depression, positive and negative affect, or valence and arousal [1, 2, 6–9, 15, 50–52, 54–56]. These pairs can often be mapped onto one another; for instance, valence and arousal can rotate into positive and negative affect [57]. The specific choice typically depends on available measurements; for example, the Young Mania Rating Scale and the Hamilton Depression Rating Scale naturally lend themselves to manic and depressive state variables. Still, whether two state variables are sufficient is an open question. Some research indicates irritability as a distinct and essential dimension for capturing mixed features [1, 53]. Others consider dimensions greater than three [58], or go so far as to model all individual symptoms [42, 59].

State variables may be either directly observed (e.g., depression scores) or latent. Recent approaches, such as those by Pulcu et al., represent mood as latent variables that unfold over time [2–4, 53]. The latent variable approach has a critical advantage: it accounts for measurement error. If mood ratings are treated as exact, models risk attributing measurement error to genuine mood variability, obscuring underlying processes [60]. Latent variable models disentangle genuine mood shifts from measurement errors, offering a clearer view of the processes driving the condition.

State variables can also be either discrete or continuous. Discrete variables assume distinct mood states, such as mania, depression, euthymia, or a mixed state, while continuous variables represent mood along a spectrum, with higher values indicating more severe symptoms. Some models might use discrete variables for simplicity. For example, the model in Prisciandaro et al. captures mood dynamics using only *nine* probabilities, describing transitions between the three mood states [4]. In contrast, continuous state variables require *infinitely* many probabilities, describing transitions between each region of mood space to another.

The choice between discrete and continuous state raises a deeper question: Do mood states like mania and depression represent fundamentally different “modes” the brain enters, or are they simply more intense manifestations of the same underlying process? Models such as the *DynAffect* framework [61] or Cochran et al.’s [54] adopt the latter view. They posit every person has a single “home base” mood to which they typically return. On this account, individuals with BP do not enter fundamentally different mood states than nonclinical controls. Instead, their mood’s home base is set to be more depressive or manic, with greater variation and slower returns to the home base [54]. This aligns with the simple, forecasting models relying only on the most recent rating [12–15, 49, 60, 62], and is consistent with Pulcu et al.’s model [2], when one interprets slower return speed as greater volatility.

An alternative theory proposes that people with BP differ from nonclinical controls by having multiple such home bases, each corresponding to distinct mood states like depression or mania [55],]. This idea is behind the forecasting methods aimed at detecting early warning signs of transitions between mood states [18–20, 25]. Although conceptually appealing, this assumption remains largely untested, and empirical evidence for distinct home bases in BP is inconclusive [19, 54, 62, 63]. While diagnostic manuals define mania and depression as separate categories, no clear biological or behavioral boundaries have been consistently demonstrated, highlighting a need for continued examination.

The Affective Ising model offers a promising middle ground, capable of representing one to four attractors, corresponding to mania, depression, a mixed state, and euthymia [63]. Though not yet applied to BP, this approach can capture critical data features, including the canonical V-shaped relation between valence and arousal [64] and the bounded nature of mood ratings [63]. This flexibility lets the model conform to patterns present in the data, rather than assuming them in advance.

Collectively, evidence suggests mood in BP is governed by two or more continuous state variables, with episodes like mania or depression forming loosely bounded regions along these dimensions. However, the field still lacks a coherent framework describing how these state variables evolve. The only widespread agreement is the important of modeling mood dynamics at the individual level, avoiding reliance on a single, generic model for all individuals with BP [33, 62].

Despite this, modeling efforts tend to fall along a continuum: from those that attribute mood fluctuation primarily to *internal dynamics* to those that emphasize *external inputs*. Internal dynamics refers to how mood depends on its own history. External inputs refers to outside influences like life events, sleep disruption, or medication changes. One extreme takes a fully deterministic stance: changes are driven entirely by internal dynamics, and what appears random is actually tiny differences in earlier states cascading into larger shifts over time [65–67].

At the other extreme are models that emphasize external inputs. These include Markov models, where external inputs are treated as random noise, often without being explicitly specified, and future mood depends only on the current mood, not the path taken to reach it. For example, consider the same person, depressed for a month in one instance, and newly depressed after weeks of euthymia in another. A Markov model would make the same forecast in both cases. The appeal of these models is largely empirical: as we saw earlier, forecasting studies suggest mood depends weakly on its past [2–5, 53, 54, 60, 61].

Between these extremes are theories proposing that mood is governed mainly by internal dynamics, with minimal influence from external inputs [46, 47, 50–52, 68–72]. These theories often assume internal processes alone, such as feedback loops or oscillatory cycles, can produce the full range of moods in BP [18, 50–52, 68–70]. Under this view, even if someone with BP were isolated from external influences, they would still experience episodes of mania and depression, purely by internal dynamics. While appealing, this assumption is difficult to test, since internal dynamics typically are not directly observable, and different models can yield similar observations. Progress is also hindered by the lack of systematic comparisons between models and the absence of agreement on core assumptions, e.g. whether mood dynamics is inherently oscillatory.

Two paths forward could strengthen these theories. The first is empirical validation: demonstrate that models based on internal dynamics improve forecasting accuracy compared to alternatives. The second is mechanistic grounding: tie internal dynamics to measurable processes. Nunes et al. [73] highlighted these concerns, cautioning that “if a model’s assumptions are not grounded in reliable and relevant facts about [BP], then the model’s ability to capture the system laws underpinning [BP] is doubtful.” They advocate for blending theory with data-driven insights.

Several initial efforts reflect these approaches. One idea is to analyze the dynamics of individual symptoms. For instance, Mesbah et al. aligned similar temporal patterns of 27 manic and depressive symptoms from 141 individuals with BP using dynamic time warping, an algorithm which involves stretching and compressing the time axis [58]. Symptoms clustered into five groups: core mania, dysphoric mania, lethargy, somatic/suicidality, and sleep. Lethargy and core mania emerged as strong drivers of other symptom groups, with somatic/suicidality and dysphoric mania more strongly influenced by them. These longitudinal findings built on earlier cross-sectional studies [59].

Another strategy connects mood to specific cognitive markers [35, 74–77]. For example, Ossola et al. linked less updating of beliefs in response to positive information to shorter periods of euthymia [75]. Zhang et al. identified dynamic brain states from resting-state fMRI data and observed that individuals with BP spent less time in a “hyperconnected” brain state than nonclinical controls [76]. Sankar et al. demonstrated that brain connectivity during emotional tasks could predict the severity of depressive and manic symptoms [35]. If tracked longitudinally, such cognitive makers could serve as windows into the internal dynamics of mood. Mirchi et al. offer a proof of principle, showing that daily mood tracked with brain connectivity patterns over a year in a single individual [78].

A final strategy is to describe external input using known biological or psychological processes, rather than as random “noise.” For example, Ebner-Priemer et al. followed 29 individuals with BP for 12 months using mood ratings and smartphone data [56]. They extracted two sets of latent variables: one for mood symptoms, the other for patterns of sleep, activity, and communication. Modeling revealed that same-day changes in sleep and activity tracked with manic symptoms. The “humble” Kalman filter, successfully applied in depression but not yet BP, offers a straightforward way to relate external inputs to known processes, providing a practical starting point for integrating these external inputs into mood models [60].

When internal dynamics or external inputs connect explicitly to measurable systems, they become viable targets for intervention. For example, Nobukawa et al. proposed using real-time brain activity data to guide targeted inputs like light or medication to stabilize sleep-wake cycles in BP, building on a model developed by Hadaeghi et al. [65, 67]. Though entirely theoretical, this line of modeling illustrates how connecting mood dynamics to measurable systems can help move toward actionable strategies.

## Conclusions and Future Directions

This review summarizes how mathematical modeling of mood dynamics are used to phenotype, forecast, and understand BP. Four core insights emerge: First, mood dynamics provides critical information about the patient beyond what is captured in summary statistics like the mean or standard deviation, differentiating BP from other mood disorders, identifying those with greater impairment in daily functioning, and measuring treatment response. Second, despite decades of effort, most forecasts of mood in BP degrade quickly in a matter of days. This appears to be an inherent feature of BP, not merely a failure of modeling. Third, mood is best represented as two or more continuous latent variables, with no clear divisions between mood states such as depression, euthymia, mania, or a mixed state. Fourth, there is little consensus on how mood evolves over time. At the heart of this debate lies a core question about whether mood dynamics are largely driven internally or externally—how much mood variability would persist in a person with BP if we could isolate them from external influences (e.g., life events, disruptions to sleep).

Alongside key insights, this review uncovered important limitations in how mood models are currently validated and applied. Remarkably, none of the reviewed studies have implemented these models in real time to inform clinical decision-making, despite their demonstrated value in phenotyping and forecasting. Given the robust retrospective support for these approaches, the next step is to translate them into tools that can actively support research and clinical decisions as they unfold. This line of work risks losing momentum unless it can demonstrate tangible value for clinical care or scientific progress.

Another limitation is that current forecasting studies are not solving the same problem. They differ in how much past data is used, how far into the future predictions are made, and how performance is evaluated. As a result, models cannot be meaningfully compared, and it remains unclear which approaches are most effective. Progress in this area would benefit from two key developments: clearly defined forecasting tasks and accessible, foundational datasets. Forecasting tasks must include clearly specified inputs, prediction horizons, and evaluation metrics, enabling models to be fairly assessed and compared across studies. The datasets should combine daily ratings of individual symptoms with passively collected signals from wearables and smartphones.

Sleep and activity data are especially promising, as they reflect core physiological processes such as circadian rhythms, and can be gathered unobtrusively and at scale. This makes them ideal candidates for evaluating how well digital signals alone can predict future mood. However, it is equally important that at least some forecasting tasks allow models to incorporate prior mood. Ignoring mood history can exaggerate the apparent predictive value of other signals, since both the predictor (e.g., poor sleep) and the future outcome (e.g., next-day depression) may simply reflect the current outcome (e.g., current depression). This creates a form of spurious predictability driven by shared temporal structure.

A final limitation is the absence of agreed-upon features of mood dynamics in BP. Without such a foundation, it is difficult to make meaningful claims about the origins of mood fluctuations. One practical starting point is to ask whether a model explains more variation in mood than competing approaches. Another opportunity is to link mood models to systems that are measurable, like circadian rhythms, and to sample these systems longitudinally alongside mood. When components of mood dynamics are both observable and tracked over time, they also become potential targets for intervention, making their role in the system clinically meaningful. 

## Key References

- Pulcu E, Saunders KEA, Harmer CJ, Harrison PJ, Goodwin GM, Geddes JR, Browning M (2022) Using a generative model of affect to characterize affective variability and its response to treatment in bipolar disorder. Proc Natl Acad Sci 119:e2202983119.

*This study distinguishes between two forms of mood variability called volatility and noise using a computational model applied to long-term mood ratings. Results show that BP is characterized by increased volatility and borderline personality disorder by increased noise*,* and lithium raises positive affect volatility rather than dampening variability.*

- Mildiner Moraga S, Bos FM, Doornbos B, Bruggeman R, van der Krieke L, Snippe E, Aarts E (2024) Evidence for mood instability in patients with bipolar disorder: Applying multilevel hidden Markov modeling to intensive longitudinal ecological momentary assessment data. J Psychopathol Clin Sci 133:456–468.

*This study uses multilevel hidden Markov modeling on high-frequency mood tracking data to uncover individual-specific mood dynamics in BP. It demonstrates that mood instability is frequent and structured even outside of acute episodes*,* challenging the traditional episode-based view of the disorder.*

- Busk J, Faurholt-Jepsen M, Frost M, Bardram JE, Kessing LV, Winther O (2020) Forecasting Mood in Bipolar Disorder From Smartphone Self-assessments: Hierarchical Bayesian Approach. JMIR MHealth UHealth 8:e15028.

*This study uses hierarchical Bayesian regression to forecast mood in individuals with BP using smartphone-based self-assessments. The hierarchical Bayesian approach yields better 1-day forecast accuracy than pooled or separate models. Their best performing model used four prior days of mood data to predict the next day’s score*,* but accuracy declined as the prediction window extended to seven days.*

- Lim D, Jeong J, Song YM, Cho C-H, Yeom JW, Lee T, Lee J-B, Lee H-J, Kim JK (2024) Accurately predicting mood episodes in mood disorder patients using wearable sleep and circadian rhythm features. Npj Digit Med 7:324.

*This paper develops models that predict future manic and depressive episodes using sleep-wake data from smartphones and wearable devices. They derived 36 sleep and circadian rhythm features which enabled accurate next-day predictions for depressive*,* manic*,* and hypomanic episodes.*

- Mesbah R, Koenders MA, Spijker AT, de Leeuw M, van Hemert AM, Giltay EJ (2024) Dynamic time warp analysis of individual symptom trajectories in individuals with bipolar disorder. Bipolar Disord 26:44–57.

*This paper uses the Dynamic Time Warp algorithm to extract shared temporal symptom relationships from BP symptoms. The results show lethargy and core mania symptoms to be a leading indicator in symptom progression.*

- Zhang X, Yang L, Lu J, Yuan Y, Li D, Zhang H, Yao R, Xiang J, Wang B (2024) Reconfiguration of brain network dynamics in bipolar disorder: a hidden Markov model approach. Transl Psychiatry 14:507.

*This study uses hidden Markov models to examine dynamic states in individuals with BP*,* representing moment-to-moment changes in brain connectivity derived from resting-state fMRI. Findings show that individuals with BP spend less time in efficient*,* hyperconnected states.*

- Malamud J, Guloksuz S, Winkel R van, Delespaul P, Hert MAFD, Derom C, Thiery E, Jacobs N, Rutten BPF, Huys QJM (2024) Characterizing the dynamics, reactivity and controllability of moods in depression with a Kalman filter. PLOS Comput Biol 20:e1012457.

*This paper employs Kalman filters to analyze mood data from individuals with and without depression. The Kalman filter framework outperforms the standard autoregressive approach and demonstrates that mood in depressed individuals is more stable and more reactive to negative external inputs compared to controls.*

## Data Availability

No datasets were generated or analysed during the current study.
